# Clinical Society of Maryland

**Published:** 1886-06

**Authors:** 


### Society Reports.

### CLINICAL SOCIETY OF MARYLAND.

### STATED MEETING, HELD APRIL 2ND, 1886.

Officially reported for the ATLANTA MEDICAL AND SURGICAL JOURNAL.

Dr. O. J. Coskery read some notes from

### A CASE OF ARTIFICIAL ANUS.

Dr. S. T. Earle called attention to a case similar to that of Dr. Coskery, reported in the London *Lancet* for May, 1885. The operation terminated fatally because of the puncture of the peritoneum. He asked if the plan advised of dissecting out the coccyx and turning back the rectum was not the preferable way of relieving these cases.

Dr. Randolph Winslow said it seemed to him that a cutting operation, the splitting of the anus to the coccyx and, if necessary, the removal of the coccyx and the bringing down of the rectum and uniting it with the anus, would have been much more satisfactory in its results and much less severe on the child than the method pursued in this case. Even if the bowel was fortunately entered by the trocar, without injuring some of the neighboring viscera or the peritoneum, the difficulty of keeping a hole, tunneled through the tissues, patent, was a very great objection to the method. As was well known the parents of a child would be likely to neglect systematic dilatation and the channel would close, as it had done in the case reported three times already. When the mucous membrane of the rectum can be attached to the anus this tendency to contraction is nearly overcome. If it is impossible to attach the rectal mucous membrane to the skin a colotomy would probably be the preferable operation.

Dr. Coskery thanked Dr. Earle for calling his attention to the article in the *Lancet.*

The course pursued by him in his operation was the one desired by the parents of the child. He gave them their choice of the operations and they united upon the least bloody one being done.

Were he called upon to do the operation again, he would adopt the plan of drawing down the rectum and uniting it to the external surface. He did not understand how Dr. Winslow could prefer a colotomy, when often by an incision two inches deep one could easily enter the rectum.

Dr. Earle, in speaking of the adhesions so often met as an obstacle to drawing down the rectum, gave as his opinion that they are not congenital, but are rather the result of manipulation with trocar and canula.

Dr. John Morris, while in the Dublin rotunda, saw two cases of congenital imperforate anus. In one there was a *cul-de-sac* leading a short distance into the soft tissues, and in the other there was but a depression at the point where the anus should have been. Both cases terminated fatally. The latter one was operated upon after the trocar and canula method, and at autopsy the bladder was found to have been punctured.

Dr. Joseph T. Smith then read a paper on

ALBUMINURIA.

Dr. J. H. Branham said that Dr. Smith's statement, that the cells covering the glomeruli had a secretory function, was contrary to what he had been taught. He took them to be only a flat epithelial covering, purely mechanical in their function. They acted, according to his ideas, as a filter through which the watery and saline elements of the urine exuded. The cuboidal cells lining the tubes he thought the true secretory elements.

Dr. I. E. Atkinson considered the comparative anatomy and physiology of the kidneys in different animals a very interesting study. For example, in birds, where there is no passage of true watery urine, the tubes are very long and convoluted, thus admitting of the re-absorption of the watery elements, and the passage of the solid constituents as solids; in fishes we see just the reverse; here there is no retention of the watery portion, and we find the tubes straight and simple, admitting of the passage of the urine just as secreted.

He believes the epithelium covering the tufts different from the lining of the tubes; he don't consider it a true secretory structure, and does think that the cuboidal cells of the tubes are the secretory elements of the kidneys.

Nitric acid and heat, or Heller's test of nitric acid alone, he thinks sufficiently accurate, for all practical purposes, in detecting the presence of albumen in the urine. Uric acid is sometimes thrown down, but this is easily recognized. A rather more convenient form, in which reagents may be carried about, he finds in the test papers prepared by Park, Davis & Co. They are handy, and he has found them tolerably accurate.

Dr. C. O. Miller referred to the experiments done upon the frog in Heidenhein's Laboratory, in which it is shown that the epithelium covering the glomeruli have a true selective action. He thinks, however, that an objection of some weight may be made by the possible degeneration of the cells, caused by the injection mass employed, and thus their true function altered.

Dr. Joseph Blum has employed the test papers referred to by Dr. Atkinson, and finds that, excepting the picric acid ones, they all deteriorate after a time.

Dr. J. H. Branham read a paper on

### PERINEORRHAPHY.

Mary Johnson, colored, age about twenty years, was confined on December 1, 1885. The position was right occipito-posterior, and the child was large.

The second stage of labor lasted for nine hours, and was finally terminated after great difficulty by the use of forceps. Notwithstanding great care exercised by a skillful operator, an extensive rupture of the parts occurred.

I first saw the patient about two hours after the labor was completed. The rupture included the perineum, the posterior wall of the vagina to the extent of two and a half or three inches and an inch and a half of the rectum. It began in the middle below and extended upward to the right.

The tear through the perineum was ragged, and the sides were trimmed before bringing them together.

Three rows of sutures were used, one in vagina, one in the rectum and the other in the perineum. Some difficulty was experienced in introducing the high vaginal sutures; otherwise the operation was easy.

Before introducing the sutures the parts were thoroughly cleansed by bichloride solution, strength one in two thousand parts of water. Small iron wire was used for sutures.

After the operation a rubber tube with a number of openings in it was introduced into the vagina, the distal end being in a bottle of bichloride solution. This acting as a syphon carried off the lochia almost entirely. Parts were disinfected twice daily by fluid passed through this tube, bichloride being used part of the time and carbolic acid part of the time. Bowels were closed by opium during first six days, liquid food being given. Patient did well, highest temperature being 102° on fifth day.

Sutures were removed on twelfth day, when good union was found, all the tear being healed except at the back part of ostium vagina and fourchette. The union was partly by first intention and partly by granulation.

What is the prognosis of primary operation in complete rupture?

Tait (page 34) says: "If the case be seen immediately after the rent has been made, stitches may be applied without any preliminary paring;" but this practice has never had a satisfactory result in my hands; for in all the cases where I have been called in to perform it, the tissues have been so bruised and the edges have been so ragged that a satisfactory union has never been effected save by a subsequent operation. It is, however, worth trying.

Others have met with little better result. "I attribute the success in this case to drainage and antisepsis, and think that unless such measures are carried out there is little chance of a good result. The vagina tends to dilate and the lochia to collect in it, when, unless some means are employed to prevent, it undergoes decomposition and prevents union."

Dr. W. Pawson Chunn—Was there any recto-vaginal fistula?

Dr. Branham—None whatever.

Dr. Chunn—What was the object of stitches in the rectum?

Dr. Branham—The rectum was lacerated for one and one-half inches, and the stitches were put there to restore this laceration. Their twisted ends were on the mucous surface of the rectum, and they remained in position for twelve days.

Dr. Chunn said his reason for asking so particularly about the stitches in the rectum, and the position of their twisted ends, was that such efforts were usually not so successful as Dr. Branham's had been. The plan usually adopted with success was to twist the wires on the mucous surface of the vagina.

Dr. Randolph Winslow thought Dr. Branham could scarcely claim much of the good result attained in his case, as due to the antisepsis, which seemed to him to be very defective, being antiseptic only when the irrigations were being practiced, with an abundant opportunity for the entrance of germs in the intervals. After considerable experience in perineorrhaphy, Dr. Winslow had discarded wire satures entirely, and now used antiseptic silk. The results were equally as good, the difficulty of introduction and the removal of stitches very much less, and the comfort of the patient much greater. In cases in which women have been operated on twice, once with wire, the second time with silk, they express themselves strongly in favor of the latter. The continuous catgut suture is probably better than either, as the necessity of removing the stitches is thereby avoided.

### FOREIGN BODY IN THE NOSE.

Dr. John M. Mackenzie related a case of foreign body in the nasal cavity of a child, the removal of which was facilitated by the use of cocaine. Dr. Mackenzie regretted to be obliged to intrude upon the Society's time by bringing before them the much-talked-over cocaine, but in this case it proved itself of so much service that he considered himself justified in calling attention to its value in such cases. When he saw the child there was a bit of bone very tightly wedged between the corrugated mucous membrane. An attempt to remove it with forceps failed. He applied cocaine to the tissues in order that its power of causing contraction might lessen the pressure and enable him thus to remove the foreign particle. This effect was produced in a few minutes, and the bit of bone was easily removed.

Dr. H. Clinton McSherry had been in the habit of using cocaine with most happy results in obstinate cases of epistaxis.

### GONORRHŒAL OPHTHALMIA.

Dr. Samuel Theobald referred to a case of gonorrhœal ophthalmia that he had recently seen. When the patient presented himself the left eye only was affected, and had been so for three days. The lids were swollen, ecchymosis was present, and the cornea was sloughing at its upper margin. Right eye was at this time entirely free from the trouble. In spite, however, of every caution the right eye took on a similar condition. Treatment was at once instituted. The eye was painted over once daily with a 15 grains to ℥ of nitrate of silver solution three times daily, with a two grains to ℥ of same drug. After each application the silver was washed away with sodium chloride solution.

Beside this, boracic acid solution was instilled every four hours, and atropia employed daily. The left eye is lost. The right eye is entirely under control. This the doctor attributes to prompt treatment ; he thinks had the right eye gotten fairly under way that it would have been destroyed.

### HEMICHOREA AND HEMIPLEGIA.

Dr. Preston related the case of a girl whom he had recently seen. She had hemiplegia on one side and hemichorea on the other. The chorea came first and was followed by the hemiplegia. He thinks the chorea was caused by a minute embolus, and the hemiplegia was the result of the consequent softening or by a second larger clot. He considered the case interesting, as probably shedding some light on the pathology of chorea.

